# An Insider Data Leakage Detection Using One-Hot Encoding, Synthetic Minority Oversampling and Machine Learning Techniques

**DOI:** 10.3390/e23101258

**Published:** 2021-09-27

**Authors:** Taher Al-Shehari, Rakan A. Alsowail

**Affiliations:** Computer Skills, Self-Development Department, Deanship of Common First Year, King Saud University, Riyadh 11362, Saudi Arabia; ralsowail@ksu.edu.sa

**Keywords:** data leakage detection, one-hot encoding, oversampling technique, insider threat detection, machine learning model

## Abstract

Insider threats are malicious acts that can be carried out by an authorized employee within an organization. Insider threats represent a major cybersecurity challenge for private and public organizations, as an insider attack can cause extensive damage to organization assets much more than external attacks. Most existing approaches in the field of insider threat focused on detecting general insider attack scenarios. However, insider attacks can be carried out in different ways, and the most dangerous one is a data leakage attack that can be executed by a malicious insider before his/her leaving an organization. This paper proposes a machine learning-based model for detecting such serious insider threat incidents. The proposed model addresses the possible bias of detection results that can occur due to an inappropriate encoding process by employing the feature scaling and one-hot encoding techniques. Furthermore, the imbalance issue of the utilized dataset is also addressed utilizing the synthetic minority oversampling technique (SMOTE). Well known machine learning algorithms are employed to detect the most accurate classifier that can detect data leakage events executed by malicious insiders during the sensitive period before they leave an organization. We provide a proof of concept for our model by applying it on CMU-CERT Insider Threat Dataset and comparing its performance with the ground truth. The experimental results show that our model detects insider data leakage events with an AUC-ROC value of 0.99, outperforming the existing approaches that are validated on the same dataset. The proposed model provides effective methods to address possible bias and class imbalance issues for the aim of devising an effective insider data leakage detection system.

## 1. Introduction

Nowadays, with the widespread usage of technology to perform many sensitive activities of an organization, security and privacy threats have increased considerably. Among these threats, insider attacks are the most dangerous and costliest types of attacks. Insider attacks are malicious acts performed by users who have authorized access to an organization’s information system. Such characteristics of an authorization have made the threats caused by insiders very difficult to detect. However, overlooking such threats may lead an organization to lose its reputation and business goals. According to IBM X-Force^®^ Threat Intelligence Index [1], the most common types of attacks are those attacks caused by insiders, which represent almost 60% of total cyberattacks.

### 1.1. Insider Threats and Their Consequences

The Computer Emergency and Response Team (CERT) defined an insider threat as “a current or former employee, contractor, or business partner who has or had an authorized access to an organization’s network, system or data, and intentionally exceeded or misused that access in a manner that negatively affected the confidentiality, integrity, or availability of the organization’s information or information systems” [2].

However, the negative acts of insiders can happen intentionally or unintentionally. Whether a malicious act was intentional or unintentional, it can cause a huge damage to an organization, such as leaking sensitive data or creating backdoors for external attackers. The authors in [3] presented three major incidents of insider attacks. The first one was against the US National Security, which was executed by one employee of the U.S. Federal Bureau of Investigation (FBI) who leaked highly sensitive data to Russian agencies. This data leakage incident caused a severe negative impact on the reputation of both the U.S and FBI. The second one was against the U.S army where one of their crew leaked a large number of top-secret documents to WikiLeaks. The last one was against Societe Generale French bank that cost an estimated loss of $7 billion due to a fraudulent act conducted by one of its employees.

Additionally, the U.S. Security Service and CERT [4] pointed out 1154 real insider incidents and categorized them into four classes (sabotage, fraud, theft and miscellaneous). They found that most of the incidents—659—were fraudulent acts that modified or deleted assets for personal gains. According to the study, the second major insider threat incidents was 189 data theft cases that stole intellectual properties of several organizations. The rest of the cases aimed at disrupting business operations which are classified as sabotage and miscellaneous. It should be noted that not all insiders’ attacks are reported due to the fact that most organizations fear from any negative impact toward their business, if the public becomes aware of their experienced incidents [5]. Therefore, the huge loss and the negative impact of insider attacks that the organizations are facing create a necessity for developing insider threat detection systems.

### 1.2. Strategies for Insider Threat Detection

Due to the reliance on using digital assets that could be stored in PCs, removable devices, emails, servers and so on, the protection of such assets from insider threats becomes a challenging problem. Since digital assets are of great importance, as their integrity is essential to the success of organizations, some organizations implemented various measures to mitigate insider threats, such as employees vetting, authentication mechanisms, training, monitoring, separation of duty, etc. [6] However, due to an authorization characteristic that the insiders have, traditional measures are effective to detect insider threats and mitigate their impacts [7].

Generally, research on insider threat detection can be divided into three areas [8]. The first area focuses on developing rule-based detection systems [9,10]. They are based on predefined rules for identifying malicious acts of insiders. A group of experts defines rules, then all behaviors of insiders are recorded and compared against the predefined rules. The types of insider threats and the domain knowledge of preventing and detecting insider threats are discussed in [11]. The limitations in rule-based detection approaches are that, there is a need to update the rules continuously through the knowledge of domain experts, and there is a big chance for the rules to be circumvented [12]. Therefore, such an approach is rigid but it can result in undesired detection performance [9,12].

The second area focuses on developing a network graph where the structure of the graph is monitored to detect any possible deviation that may refer to malicious behaviors [13]. In such approaches of graph-based insider threat identification, the value of the data and the relationships among the data are analyzed. The relationships among the data are represented by edges that connect the nodes in a graph. By analyzing its properties, the relationships of particular nodes within graphs can determine malicious insider acts.

The third area focuses on applying machine learning (ML) techniques for detecting malicious acts of an insider [14]. In ML, a machine learns algorithms on training data to perform specific tasks for performance optimization [15]. In the area of insider threats, ML is utilized to create models that automatically identify different malicious acts. Since ML uses the data to learn and update the algorithms constantly, its detection performance can be more accurate and stable.

This paper employs ML to detect data leakage acts that could be carried out by malicious insiders. The detection of data leakages is extremely significant, due to the great harm that can occur if the sensitive data is exfiltrated from the systems of an organization. Therefore, this paper proposes an insider data leakage detection model for the aim of assisting cybersecurity analysts to deal with the problem and find out the suitable solution. The contribution of the paper can be summarized as follows:

- It proposes a unified model to detect a data leakage threat that could be carried out by a malicious insider during a sensitive period, before his/her leaving to an organization.
- It addresses the bias issue that could happen due to an inappropriate encoding process, while detecting the insider threat, utilizing the one-hot encoding method.
- It handles the class imbalance problem of the dataset by applying the SMOTE technique.
- It implements the most well-known ML algorithms (LR, DT, RF, NB, k-NN and KSVM) for detecting data leakage acts. The performance of the applied algorithms is compared, for a better view of the optimal detection model.
- It applies significant metrics for evaluating the performance of ML algorithms (precision, recall, F-measure and AUC-ROC value), as they consider the bias problem of the encoding process and the imbalanced classes of the CERT dataset.

The rest of the paper is structured as follows: Section 2 reviews the related work. Section 3 illustrates the proposed model, the employed dataset, the pre-processing steps and the applied ML algorithms. Section 4 discusses the experimental evaluation and the obtained results. Section 5 discusses and compares the results of the applied model compared to with the existing approaches. Finally, the conclusion, limitations and future work are deliberated in Section 6.

## 2. Related Work

Insider threat detection is a challenging problem for research communities and cybersecurity companies. During the last decades, considerable research efforts have been made to address this problem. The U.S. Security Service and CERT issued common guidelines to detect and alleviate insider attacks in organizational environments [4,16]. They include some best practices for organizations to follow in order to prevent and detect various insider threats. Several case studies of organizations failures, with respect to insider threats, can be found in [4]. The studies in [17,18,19] reviewed the literature of the insider threat detection area from different perspectives. For example, cybersecurity issues related to insider threats, such as advanced persistent threats and malware, are reviewed in [17], while a classification of the insider threat research is demonstrated in [18]. The study in [19], categorized and compared different empirical techniques for insider threat detection in terms of 10 significant factors (e.g., datasets, feature domains, classification techniques, simulated scenarios, performance and accuracy metrics, etc.). It highlights the factors that reflect the methodology and performance of reviewed approaches from various empirical perspectives. The survey in [20] reviewed the deep learning approaches for insider threat detection and illustrated the commonly-used datasets in the field. It showed how deep learning models can improve the detection performance compared to traditional machine learning algorithms. On the other hand, it presented the limitations that the deep learning models can face in the insider threat detection task (e.g., adaptive attacks, lack of labeled data, etc.). Several challenges were discussed, and future research directions were suggested to boost the insider threat detection performance of deep learning models. The study in [21] surveyed general aspects of insider threats on the IoT based models with respect to private and public sources. They observed the data sources on the IoT environments utilizing various characteristics, such as application layer, perceptual layer and network layer. They categorized the data sources, objectives and methods of data features for each layer. The findings of the study presented that within the IoT environment, the perceptual layer data sources are less suitable than data sources from application and network layers. The study presented the limitations of using the data features from application, perceptual and network layers in the IoT environment. The survey in [22], aimed to review notable insider threat detection approaches from different aspects: investigated behaviors, machine learning methods, datasets, detection techniques and performance metrics. It also presented a classification of recent insider threat types, access methods, level, motivation, insider reporting, security assets, etc. It analyzed different insider threat incidents that provide statistical information of malicious insiders. The survey presented the challenges in the field of insider threat and highlighted some recommendations that can help in overcoming such obstacles. 

Nowadays, with the massive amount of data that organizations deal with on a daily basis, the security of such data has become a challenging problem. A promising approach to overcome such problems is by utilizing ML based solutions [23,24]. From massive data, ML based solutions can automatically learn, identify patterns and classify possible malicious acts as shown in [25,26]. The authors in [27] utilized a detection algorithm based on a graph to detect possible insider threats. In [28], a Bayesian Network-based model was proposed to identify insiders’ malicious acts. The authors identified indicators of malicious acts via an empirical study that could enhance the design of insider threat detection systems. The authors in [29] utilized graph models and presumed an approach of incremental learning under streaming data to detect insider threats. This was done through creating quantized dictionaries of patterns for each chunk of data. If the data had a large edit distance from all patterns in the dictionaries, it was detected as an anomaly. In other anomaly detection approaches, the sequences of insiders acts are modeled to detect any deviations from such sequences [30,31]. The authors in [31] employed a Hidden Markov Model to model the sequences of normal users acts in a weekly basis, thus, any anomalous acts are detected they were considered as potential insider threats. The Anomaly Detection at Multiple Scales (ADAMS) project of Defense Advanced Research Projects Agency (DARPA) [32] aimed at identifying anomalous acts in huge datasets for detecting and preventing insider threats. ADAMS’s project supported many insider threat detection systems [30,33,34,35]. The study in [30], introduced a visual language for describing anomalies, while the authors in [32] followed a hybrid approach to combine anomaly detectors to detect two classes of insiders: those who blend in malicious insiders and insiders who have unusual change in behaviors. The authors in [36,37], introduced approaches of Masquerader detection utilizing anomaly detection in the behavior of user search and file access. In [38], a holistic insider threat prevention framework was proposed. It considered three modes of insider threat countermeasures i.e., pre-countermeasures, in-countermeasures and post-countermeasures. Such countermeasures employed technical, psychological, behavioral and cognitive measures that span from the pre-joining of an insider to an organization till after they leave. The authors illustrated their approach utilizing three real-world insider threat cases. In [39], the authors introduced a cyber-security culture framework for detecting malicious and unintentional insiders by focusing on human factors. Their framework considered various indicators in the insider threat detection (personal, behavioral, cultural and technical) to assist in detecting possible security threats arising from privileged users. They also linked existing insider threat classes with specific security domains to provide an evaluation methodology of the main contributing criteria. A framework for insider threat detection, based on persistence analysis methods, was introduced in [40]. The authors utilized Cox proportional risks for the aim of predicting insider threat events with more accuracy. Various features were employed (e.g., insider acts, logon data, psychometric assessments, etc.). The aim of the proposed framework was to address the challenge of predicting insider threat incidences considering the estimated time of occurrences within limited resources. In [41], a framework for insider threat detection was proposed utilizing hardware and system-based level. The framework detected an insider threat by analyzing the behavioral dynamics of USB devices before malicious insiders modify the data in a corrupted OS. A decision tree classifier was employed to detect anomalies of USB device behaviors. The experimental results showed that the framework detected anomalous USB acts with a ROC AUC of 0.99. 

In [34], various ML algorithms were utilized to detect anomalies and early ”quitter” indicators, which both indicate potential insider threats. The ML algorithms are examined for their capabilities to detect insider threats. For classifying conditions of non-stationary insider behaviors, stream online learning approaches were utilized. Under streaming conditions, a common technique to support threat detection is by utilizing weighted average [29,42]. The authors in [42] introduced an insider detection approach based on deep neural networks (one model for the organization), or (one model per user) using recurrent neural networks. The authors in [43], introduced a system to detect insider threats utilizing scalable supervised and unsupervised learning algorithms on a fusion of heterogeneous data streams. In [44], an approach for detecting insider threat is proposed utilizing programming algorithms that was evaluated under constant and non-constant behavioral assumptions of insiders. In [45], a machine learning based system was proposed to detect insider threats utilizing user-centered analysis. The aim was to detect malicious insiders by analyzing insider acts on multiple data granularity levels simulating realistic scenarios. The experimental results showed that the system was able to detect malicious insiders with an accuracy of 85% and a false positive rate of 0.78%. In [46], a deep learning-based insider threat detection model was proposed. It employed the Dempster-Shafer theory to detect accidental and intentional insider threats through the real time communication network within an organization. The anomalous behaviors of network patterns were detected utilizing the long short-term memory (LSTM) and multi-head attention mechanisms. They enhanced the detection performance of their model by updating the certainty utilizing Dempster’s conditional rule. Data theft, leakage and unauthorized sharing attacks are straightforward for insiders as they have authorized access, thus, in [47] a scheme for cybersecurity was proposed to detect insider threats based on anomalous behavior of insiders. In [48], an approach for evaluating insider threat detection methods was proposed. Three modules of email-based active indicators were assessed to differentiate between malicious insiders and benign users. The active indicators included the exploitations of gathering information, the avoidance of detection, or maintaining hypervigilant security awareness. Their findings indicated that the active indicators can be evaluated utilizing reality games to assess their effectiveness in a real-world environment. They also found that by utilizing some kinds of active indicators the insider threats could be detected in real-world workplace settings.

Differing from the existing approaches, which focused on detecting insider threats without considering significant factors such as the possible bias and data imbalance issues, our work aims to develop an ML model for insider data leakage detection by considering the possible bias problem of an inappropriate encoding process and the imbalanced classes of a dataset. To investigate that, it applies different techniques (label encoding, feature scaling, one-hot encoding and SMOTE) over the most well-known ML algorithms. The CERT insider threat dataset [49] is utilized for evaluating the proposed model. Different evaluation metrics are also implemented to demonstrate the practical performance of the model.

## 3. Methodology

**Figure 1:**
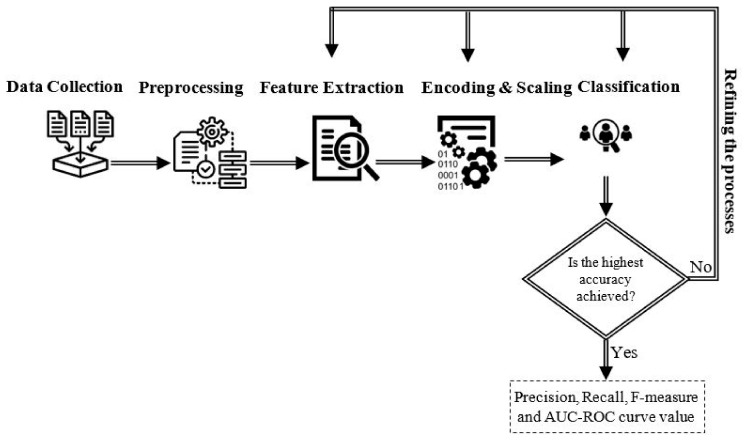
An overview of the system.

The aim of this research is to investigate the ability of various ML algorithms for detecting insider data leakage incidents in an organizational environment. To accomplish this, an insider data leakage detection model is developed. An overview of the model is illustrated in Figure 1. It consists of several modules: data collection, pre-processing, feature extraction, encoding and scaling, and classification. The following sections provide a detailed description of the system’s modules.

### 3.1. Data Collection

In the first stage, activity logs of insiders should be captured from various sensors within an organization. One of the major challenges for the research community of insider threat is to obtain a real-world corporate dataset, due to security and privacy concerns. Therefore, the Software Engineering Institute at Carnegie Mellon University created the CERT dataset [49], which is utilized widely by the insider threat research community. It is a “free of privacy and restriction limitations” [50] to allow researchers on insider threat topic for experimenting and assessing their proposed solutions. Therefore, the CERT dataset is employed to validate the performance of our model. It contains activities logs of insiders that are generated from a simulated organization’s network with sophisticated models [42]. The dataset version “R4.2.tar.bz” has been utilized in this research, as it is the “dense needle” version with a reasonable amount of red team scenarios. It consists of 1000 insiders’ activity logs recorded for over 17 months. It includes several log files (logon/off, file operations, HTTP, email and removable device). A brief description of the dataset files is provided in Table 1.

In addition to the provided dataset files, different insider threat scenarios are also defined. Our study focuses on detecting a sensitive insider threat incident where a data leakage attack is executed by a malicious insider before his/her leaving from an organization. A description of the attack scenario is briefed as “A user who did not previously utilize removable drives. Then, he starts logging to an organization system after normal working hours, uses removable devices, uploads data to wikileaks.org, and leaves the organization shortly thereafter”.

### 3.2. Pre-Processing

Data pre-processing is an essential part of data analysis, which aims to remove unwanted data noise and thereby refining the data features of interest. Moreover, the choice of optimal pre-processing methods can strongly influence the analysis results. In this study, various pre-processing methods are applied (e.g., aggregation, refinement, extraction and encoding of most relevant data features). Initially, the logs of insiders’ activities are stored in five separated files (logon/off, removable device, http, email and file operations). So, it is required to integrate diverse files into one homogeneous file and extracts the data features that represent malicious acts of an insider. The data files of insider acts are integrated based on data fields that reflect the insider data leakage scenario. Table 2 presents the data fields and variables of the combined dataset.

However, when the dataset files are collected in a real-world environment, some data fields may contain impurities (e.g., missing data). This is common, as the dataset is collected through different sensors which are not always working as expected. The data quality is a major concern in the classification process, so the combined files are ensured to not contain null values.

### 3.3. Feature Extraction

Once dataset files are aggregated, the feature sets are selected to represent the threat scenario of study. It is not practical to involve everything in the dataset, as the inclusion of irrelevant features of data may generate noise and degrade the performance of the model. Thus, the extraction of the most relevant features is significant to optimize the performance of ML classifiers.

The dataset includes separate files each containing so much data. So, the decision about which one to keep and which one to remove is done based on the potential threat features of the above-mentioned scenario. The relevant features include: number of logins outside regular working hours; number of times the removable devices are used outside office hours; number of times “wikileaks.org” website is visited. The extracted features and their description are presented in Table 3.

As the relevant features are extracted, they need to be encoded for ML detection models.

### 3.4. Encoding

As most ML models understand integers “not text”, converting categorical variables “text strings” into numerical ones is a necessary step, such that an ML model is able to compute the correlation between them and make the correct predictions. The extracted features contain categorical variables provided as text strings. Table 4 shows the data type of the extracted features.

Therefore, the variables of extracted features have to be encoded before feeding them to ML models. The inappropriate encoding of features’ variables can cause ML models to misinterpret the correlation between them. For instance, the “vector” feature involves logon, device and http variables. These variables can be encoded as (logon = 0, device = 1 and http = 2). However, if they are encoded like this, an ML model could understand that there is an ordering relationship between them. In fact, this is not the case, as the ordering relationship between such variables is not the focus of interest. Thus, to avoid such misinterpretation, we consider the encoding process for the variables of features based on whether they are ordinal or categorical.

The dataset features include a combination of ordinal and categorical data types. The categorical variables can be addressed using multiple encoding techniques. In our study, we apply label encoding and one-hot encoding techniques. In the label encoding, each categorical variable of features is assigned a unique integer. This can create the bias in the encoded variables and classification misinterpretation. We apply this method as a baseline for our proposed model. The results of applying the label encoding is illustrated in (Section 4.1). On the other hand, the one-hot encoding technique is applied. It is a popular encoding method to utilize when processing datasets containing categorical variables [51]. Therefore, we employ this technique in the insider data leakage detection. The one-hot encoding contains binary vectors that turn variables of categorical features the numerical values (0 or 1). The details and results of applying the one-hot encoding technique is presented in (Section 4.2). For ordinal data features “Timestamps”, they are encoded by converting them into Unix Epoch Time format. This makes it easier for computer systems to manipulate and store dated information than conventional date systems [52]. Table 5 shows the result of encoding the feature matrix for the classification process.

### 3.5. Classification

Once the raw data is pre-processed and the relevant features are extracted and encoded, they are ready for ML algorithms to ingest them. In this section, we present the most widely used ML algorithms that we implemented in this research. Brief descriptions of them are presented as follows.

Logistic regression (LR): It is a linear classification model that utilizes a logistic function to model a binary dependent variable based on many independent variables. In this research, the independent variables are represented by various activities of insiders (e.g., logon/off, webpage visits, connecting/disconnecting removable devices, etc.). The binary dependent variable represents whether an insider action is malicious or not. The logistic regression algorithm has two main advantages [51]: first, it is highly interpretable as a linear model; second, it returns the probability of an input vector that belongs to a class which facilitates the prioritization of the most suspicious activities of an insider.

Random forest (RF): It is an ensemble process that trains several decision trees in ensembles to provide a single class label for each ensemble [53]. Given “T” training ensembles that are described in terms of F features. The RF decision trees are defined by: (1) choosing a random subset “T” from the training ensembles; (2) recognizing a random subset “F” from their features; (3) each of the “F” selected features are utilized to parameterize a new node in the decision tree by using a Gini index [54]. Such processes are repeated to establish specified numbers of decision trees of insiders. The properties of RF have been shown make robust predictions [55].

Support vector machines (SVM): This algorithm represents a class of supervised ML. Given a set of variables that belong to any two classes, the objective of the SVM algorithm is to find an optimal hyperplane that separates the two classes of variables. It is designed to minimize the misclassifications of training and test samples. It is one of the most widely used ML algorithms in many applications, such as speech recognition, image classification, intrusion detection and so on. The characteristic that makes SVMs suitable to be employed in cyber security is that, its latency is very low, which provides high performance on a dataset with realistic scale and complexity [14]. So, it is applied in this research for an attempt to increase the accuracy of the detection process while classifying insiders’ normal actions as opposed to malicious ones.

Naive Bayes (NB): It is also known as Bayesian classifiers that allocate a class to a given sample defined by its feature vector. Assuming that P(X|C) = $\Pi_{i=1}^{n}$P(Xi|C) where X = (x1, x2, …, xn) are independent feature vectors and C is a dependent class. Despite this idealistic assumption, the resulting classifier is effective in practice when competing with other sophisticated techniques [56]. It has also proven in [57] that NB is an effective classifier in various real-world applications (e.g., medical diagnosis, text classification, etc.).

K-nearest neighbors (KNN): It is also known as nearest neighbor classification. The idea behind the KNN is that the nearest data points to a target data point “x” for which we look for a class, provide useful information. It allocates a class label of the common K-nearest data points in the dataset. The KNN is an outstanding classifier for datasets with low dimensions and large size of training set, but in case of high-dimensional datasets an extension of the KNN should be applied [58].

Kernel SVM (KSVM): The SVM algorithm employs a set of mathematical functions that are defined as kernels. The SVM is a linear classifier that classifies feature vectors linearly, but they might not be linearly separable [59]. Thus, the KSVM trick is used to overcome this issue by mapping a data input into a high-dimensional feature space using kernel functions. The SVM utilizes various kernel functions (e.g., polynomial, radial basis function (RBF), sigmoid, etc.). The performance of SVM classifiers is based on a selected kernel function. Such kernel functions (KSVM) are applied for diverse classification tasks in [59,60,61].

Decision tree (DT): It is also known as C4.5 algorithm [62]. It creates a tree based on the if-then rule at each node of the tree using the concept of information entropy. It requires a labelled training data for at least two classes. The data is split into two subsets at each node of the tree. This is done by selecting a feature and a data point that give the highest normalized information gain. Then, the algorithm is repeated on remaining subtrees. Each decision at a node of the tree is characterized as a rule on an input space, making DT one of the most commonly used algorithms due to its interpretability and efficiency [63].

We provide a brief overview of the applied ML algorithms in cyber-security applications. More details on their foundations can be found in [64]. The ML algorithms that we apply in (Section 4) provide several parameters that can be tuned. In our experiments, we utilize the default parameters of the applied ML algorithms.

### 3.6. Performance Metrics

The ML algorithms are employed in our model to classify whether an insider event is a data leakage event or not. The performance of the applied ML algorithms is assessed utilizing various evaluation metrics. However, the accuracy metric is an inappropriate performance measure for ML classifiers when dealing with an imbalanced dataset [65]. In the imbalanced classes of a dataset, an ML model places more weight on majority classes than on minority classes, which makes it difficult for a classifier to perform well on the minority classes [66]. In the CERT insider threat dataset that we employed in our study, the non-malicious acts are much more than malicious ones. Therefore, in our model we utilize the evaluation metrics (confusion matrix, precision, recall, f-measure and AUC-ROC value), as they provide a better insight of the classification when dealing with imbalanced classes [67].

Confusion matrix (CM): It is a table that shows a number of acts that are detected correctly and incorrectly. The CM is used commonly to compute the performance metrics of classification algorithms [68]. As shown in Table 6, the rows represent the actual malicious and non-malicious instances, while the columns represent detected malicious and non-malicious instances.

**Figure 2:**
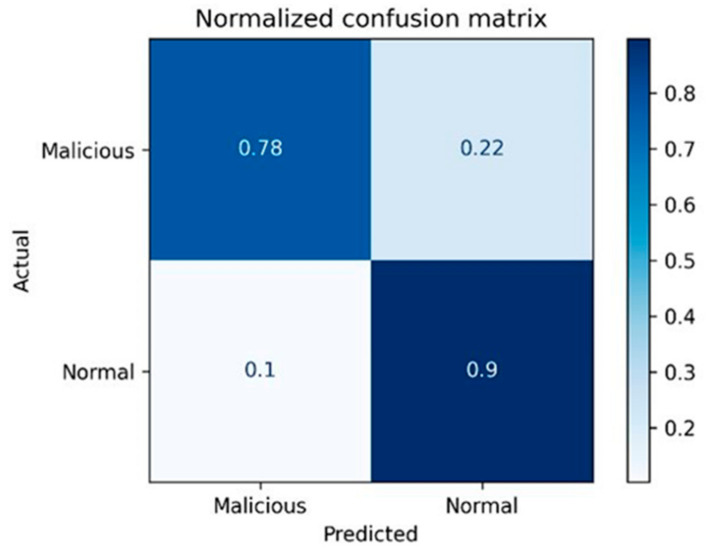
Result sample of applying CM for evaluating the insider data leakage detection using NB classifier.

As presented in Table 6, the CM provides valuable information TP, FP, FN and TN. The TP represents the number of malicious instances that are detected correctly as malicious, while the FP represents the number of non-malicious instances that are detected wrongly as malicious. On the other hand, the FN represents the number of malicious instances that are detected wrongly as non-malicious instances, while the TN is the number of non-malicious instances that are detected correctly as non-malicious instances. Figure 2 shows an example of implementing a confusion matrix for detecting data leakage instances utilizing the NB classifier.

Figure 2 shows a sample of applying the CM for evaluating the ML models in detecting insider data leakage acts. It displays the percentage of TP, FN, FP and TN utilizing the NB classifier. From the CM, more concise evaluation metrics are implemented (Section 4), which are briefed as follows.

Precision (P): The precision measures the percentage of malicious and non-malicious instances that are detected correctly. It shows how accurate is the model. It is calculated by dividing the TP by all acts that are detected as malicious ones (TP and FP).

P = TP/(TP + FP)(1)

The P measures the exactness of the model. Low precision indicates a high number of FP.

Recall (R): It measures how good the model in detecting all the positives. It is measured by dividing the TP by all acts that are detected correctly (TP and FN).

R = TP/(TP + FN)(2)

The R is known as the sensitivity or true positive rate (TPR). It measures the completeness of the model, where the lowest recall score indicates the highest number of FN.

F-measure: This metric calculates the weighted average of precision and recall.

F-measure = 2(P × R)/(P + R)(3)

The F-measure is defined as the harmonic mean of the precision and the recall.

Area under Curve- Receiver Operating Characteristic Curve (AUC-ROC): The AUC-ROC curve value score is one of the most commonly used metrics to evaluate classification models. It represents the probability of the model for classifying observations from two classes. The more AUC-ROC value is, the more quality of a classification model we have. It is counted utilizing the following equations:

Specificity = TN/(TP + TN)(4)

Sensitivity = TP/(TN + TP)(5)

AUC-ROC curve value = Sensitivity/1 − Specificity(6)

where the TN is the true negatives and the TP is the true positives. The specificity is the true negative rate (TNR), while sensitivity is the true positive rate (TPR). In Section 4, all the aforementioned evaluation metrics are applied to give more insight on the performance of our model. 

## 4. Experimental Evaluation

In this section, we present the evaluation of the proposed system, the experimental settings and performance metrics. The implementation of the proposed model is executed utilizing the Python 3.9.0 programming language with TensorFlow [69] backend, the machine learning platform from Google. Python is one of the most widely used programming languages by developers and scientists. This is due to its richness with several open-source libraries (e.g., Pandas, Numpy, Scikit-learn, etc.), as well as its support for a large variety of ML algorithms. Thus, it is employed to verify the effectiveness of the system for detecting insider data leakage acts by considering their comprehensive contextual information. The HP system with Microsoft Windows 10 is utilized, with a processor of 64 bit with Intel(R) Core (TM) i5-4590S, CPU 3.00 GHz and Memory RAM of 8 GB. The CERT insider threat dataset [49] is utilized to validate the proposed model. A series of experiments are conducted to preprocess the raw data, build ML models and plot the results. Initially, the raw data of insiders’ acts (logon/off, http visits, removable devices, etc.) are preprocessed to make them ready for the ML algorithms to ingest them.

**Figure 3:**
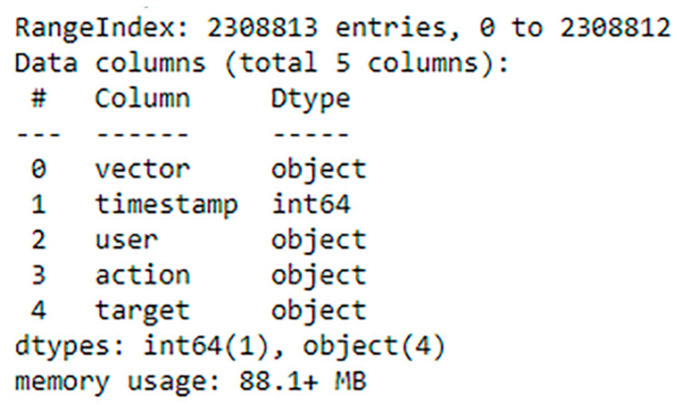
The meta data of extracted features.

The CERT dataset, in its original form, is separated into multiple files (logon.csv, http.csv, email.csv and device.csv). Thus, we consolidate them into one single file. Then, the relevant features that reflect the scenario of insider data leakage acts are extracted. Figure 3 shows the meta data of the extracted features (number of entries, columns, data types, etc.).

The performance of the model does not depend only on the selected ML algorithm, but also on how different data types of features are encoded and scaled before feeding them to ML models. Therefore, the model is evaluated utilizing different techniques label encoding, feature scaling, one-hot encoding and SMOTE. Such techniques are implemented using Scikit-learn [67], the most useful library for ML in Python. It is the ML toolkit that provides several tools for different aspects of ML, e.g., preprocessing, model selection, regression, classification, etc.

The matrix of features is divided into the training set and test set. The recommended size of splitting the dataset is 80% for the training set and 20% for the test set. Table 7 presents the results of splitting the matrix of dataset features.

Once the matrix of features is split into training set and test set, different ML algorithms are applied. The applied techniques (label encoding, feature scaling, one-hot encoding and SMOTE) in the proposed insider data leakage detection model is analyzed to evaluate their performance over popular classifiers, namely logistic regression (LR), decision tree (DT), random forest (RF), Naïve Bayes (NB), k-nearest neighbors (k-NN) and kernel support vector machines (KSVM). The performance of the different classifiers is assessed using the widely used metrics, namely precision, recall, F-measure and AUC-ROC value. The results of applying label encoding, one-hot encoding and SMOTE Techniques are illustrated in the following sections.

### 4.1. Label Encoding and Feature Scaling

Label encoder is a part of the Scikit-learn library in Python, and it is used to convert categorical data into numbers, so that the ML models can understand the correlation between various variables of features. The encoding converts the categorical data into a form that can be understood by the computer. There are different techniques to encode the categorical data, so in our study we utilize two, namely the label encoding and one-hot encoding. The label encoding technique is applied on ML algorithms in two methods: actual, where the label encoded features are fed into ML models without scaling; and scaled, where the variables of features are scaled utilizing standard scalar. The standard scalar is the process of scaling all variables of features to ensure that they are all taken the same scale (0–1). This is to avoid some features to be dominated by others in such a way that the dominated features could not be considered by some ML algorithms. Thus, the feature scaling is applied utilizing the following equation.

x scaled = (x − mean(X))/(SD (X))(7)

Each value x of a feature X is subtracted by the mean of all values of that feature, and then divided by the standard deviation of all features’ values. This will put all values of a feature on the same scale. Table 8 shows the results of applying label encoding technique utilizing the two methods (actual and scaled).

Table 8 presents the results of applying different ML algorithms in a variety of experiments (label encoding with and without feature scaling). The highest results of recall and F-measure were obtained by the DT algorithm with 74% and 71%, respectively. It is observed that there is a significant improvement when applying scaling methods over KNN and KSVM algorithms in terms of precision, recall and F-measure compared to other ML algorithms. For example, the precision score was improved from 13% to 84% when applying the scaling method over the KNN algorithm. On the other hand, the results show a slightly lower precision score (from 0.60 to 0.58) when applying the scaling method over the RF algorithm. Noticeably, when applying the DT algorithm, there was not any change in the obtained results whether the scaling method is applied or not. The same for the RF algorithm when Recall and F-measure metrics are utilized.

**Figure 4:**
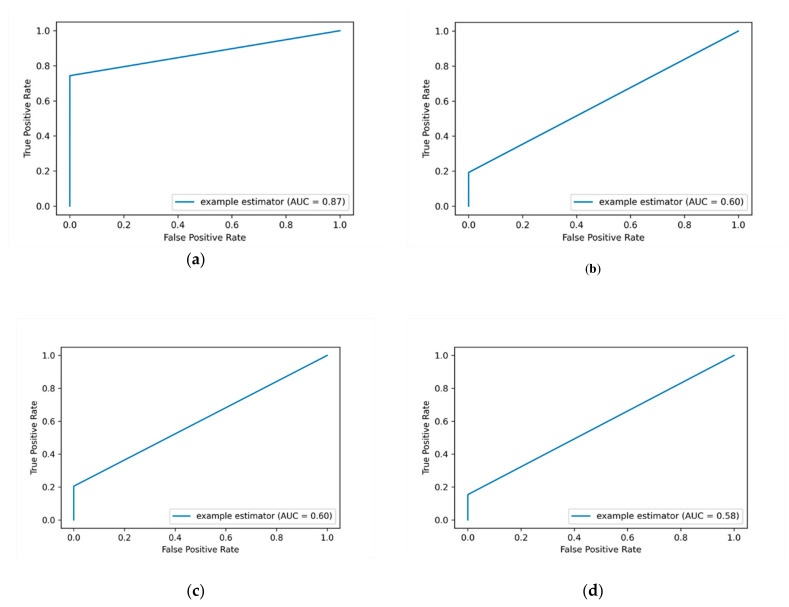
The average AUC-ROC curve value generated by applying label encoding and standard scaling. The (**a**) plot shows the AUC-ROC curve value of applying DT algorithm, and the (**b**) plot shows the AUC-ROC curve value of applying RF algorithm. The (**c**) plot shows the AUC-ROC curve value of applying KNN algorithm, while the (**d**) plot shows the AUC-ROC curve value of applying KSVM algorithm.

The ROC curve is a graphical representation that is used to plot the true positive rate (TPR) against the false positive rate (FPR). The AUC-ROC value is calculated by considering the area under the AUC-ROC curve between the range (0 to 1). The higher the TPR is the better the ML classifier is. When applying feature scaling for the labeled encoding data, an improvement of AUC-ROC value over some ML classifiers was attained. For example, the AUC-ROC value is improved from 51% to 60% when applying the KNN algorithm, and from 50% to 58% for the KSVM algorithm. Figure 4 depicts the AUC-ROC curves of applying label encoding and standard scalar for detecting insider data leakage instances.

In this work, the performance of the insider data leakage detection model is also measured utilizing AUC-ROC curve metric. It depicts the relationship between the true positive rate (TPR)/detection rate (DR) and the false positive rate (FPR) under different decision thresholds. The AUC-ROC value summarizes the AUC-ROC curve in a single numerical metric to compare between different ML algorithms. Figure 4a shows that the DT algorithm has the most top-left-side AUC-ROC curve with an AUC-ROC value of 87% compared to other algorithms. This is followed by the RF algorithm with an AUC-ROC value of 60% and the KSVM algorithm with an AUC-ROC value of 58%. It is observed that the detection results of applying label encoding method are improved when utilizing the standard scaling over the DT algorithm. Overall, the results show that when the label encoding method is applied, the detection results are low. Thus, we put this method as a baseline to evaluate our model over the One-hot encoding and SMOTE techniques as presented in the following sections.

### 4.2. One-Hot Encoding

The type of encoding method plays a major role in the performance of the classification process. One-hot encoding is a common technique for handling categorical data [70]. It is the process of converting categorical variables into a format that allows ML models to do a better job in detecting insider data leakage instances. It simply highlights the presence of features’ variables to avoid the misinterpretation of the correlations between independent variables. One-hot encoding is an effective encoding scheme for addressing classification tasks [71]. The previously applied label encoding method is easier than the one-hot encoding method, but with label encoding some ordering issues can arise as some numerical values may be misunderstood by some ML algorithms. To address such ordering issues, the one-hot encoding technique is employed. With one-hot encoding, each categorical value is transformed into a new column, and the label values are converted to a digital form either (1 or 0). An example of applying one-hot encoding process on dataset features is shown in Table 9. 

As shown in Table 9, the one-hot encoding is carried out for insider dataset features named “Insider Acts” and the label values of the features are “Login”, “USB”, “Website” and “Logoff”. For example, when an insider instance relates to Login, then it is assigned with “1”, otherwise it is assigned with “0”. Similarly, the same one-hot encoding process is applied for all other instances. At the end of the one-hot encoding transformation process, we ended up with a feature matrix of (1847050, 5481) for the training set and (461763, 5481) for the test set. Table 10 shows the results of applying one-hot encoding technique on different ML models.

As presented in Table 10, the results show significant improvements when applying the one-hot encoding method compared to the label encoding method (Section 4.1). As mentioned in the performance metrics (Section 3.6), the precision metric evaluates the exactness of the model. Thus, it is noticed that the LR and KSVM algorithms achieve the highest precision score. This means that they are able to correctly detect insider data leakage instances (true positives) with 100%. As shown in Table 10, the results of applying the one-hot encoding method provide more reasonable performance of the model compared to the results of label encoding (Table 9). This is because no zero scores are obtained for precision, recall and F-measure metrics.

**Figure 5:**
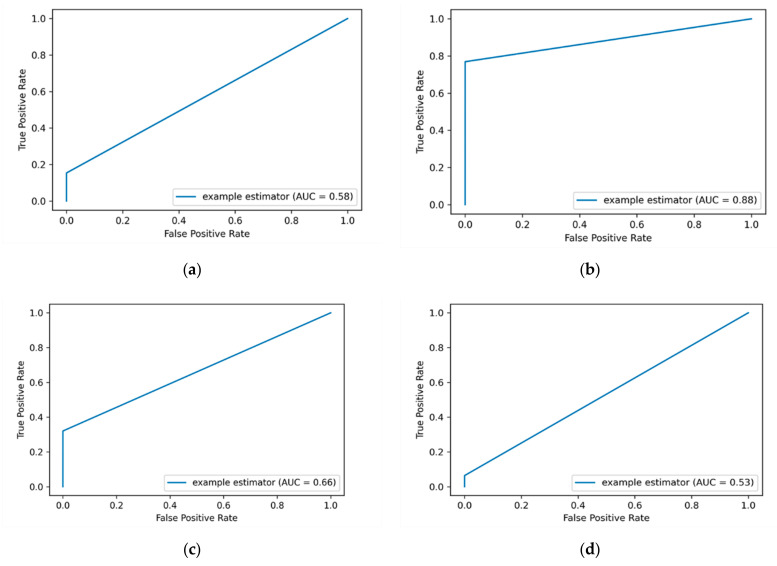

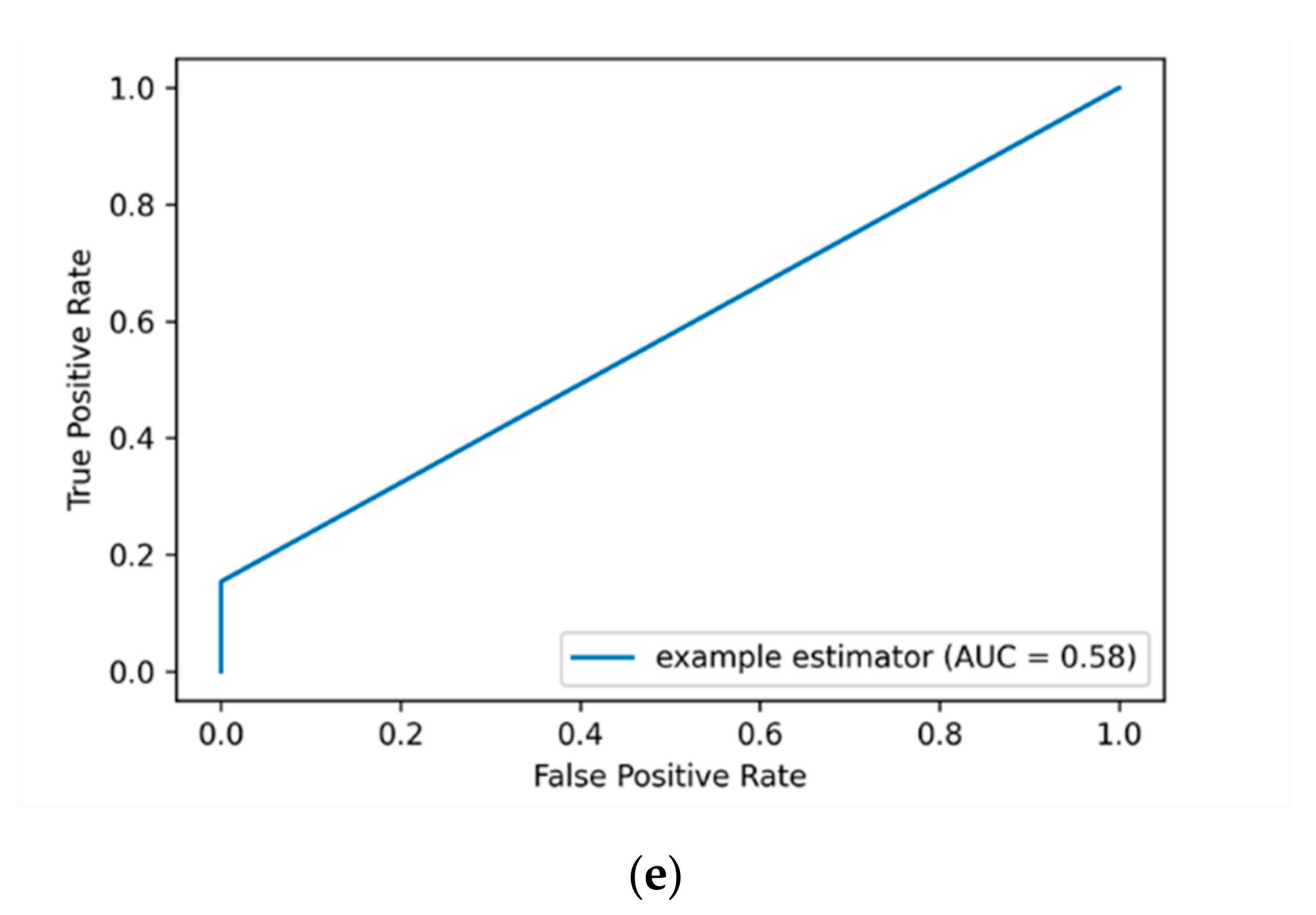
The average AUC-ROC curve values generated by applying the one-hot encoding method. The (**a**) plot shows the AUC-ROC curve value of applying the LR algorithm, while the (**b**) plot shows the AUC-ROC curve value of applying DT algorithm. The (**c**) plot shows the AUC-ROC curve value of applying RF algorithm and the (**d**) plot shows the AUC-ROC curve value of applying the KNN algorithm. The (**e**) plot shows the AUC-ROC value of applying the KSVM algorithm.

In order to get the value that tells us how good the model is in classifying the malicious instances from non-malicious ones over a one-hot encoding method, the AUC-ROC value is calculated. The more top-left the curve is the higher the area and hence the higher AUC-ROC value is. The average AUC-ROC curve values of applying one-hot encoding are presented in Figure 5. It shows that the DT ML algorithm still performs the best with an AUC-ROC value of 88% compared to other ML algorithms.

The DT algorithm achieves the best AUC-ROC value of 88% compared to other ML algorithms, this is followed by the RF algorithm with an AUC-ROC value of 66%. The lowest detection performance is obtained by the KNN algorithm with an AUC-ROC value of 53%. It is observed that both the LR and KSVM algorithms got the same AUC-ROC value of 53% over the one-hot encoding method.

### 4.3. Synthetic Minority Oversampling Technique (SMOTE)

With binary classification problems like “insider threat detection”, the class imbalance is a scenario of having an unequal distribution of classes in a dataset (e.g., the number of non-malicious instances is very large compared to malicious instances). The utilized dataset has high class imbalance with a number of “346” malicious instances compared to “2308467” non-malicious instances. So, if the imbalanced classes in a dataset are not treated properly, the performance of the classification model can be degraded. In such a situation, the model can correspond with the majority class and treat the minority class as noise. Furthermore, it might neglect the minority class, and the evaluation metrics can be biased. Therefore, we apply the SMOTE technique [72] to address the class imbalance of the dataset. The algorithm of the SMOTE technique works as follows:

1. Select a data point from the minority class as an input.
2. Find its k nearest neighbors as an argument for the SMOTE () function.
3. Select one of these neighbors and place a synthetic data point anywhere on the line which fits both the data point and its neighbors.
4. Repeat the steps until the dataset is balanced.

The results of applying the SMOTE technique are presented in Table 11.

**Figure 6:**
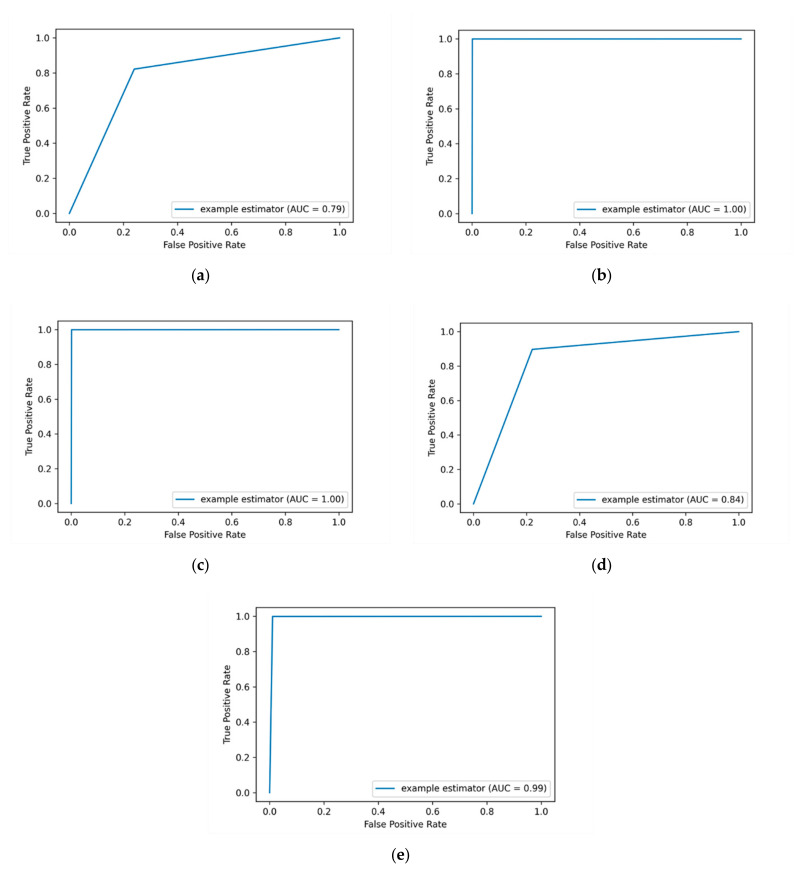
The average AUC-ROC values of applying SMOTE technique in the detection process. The (**a**) plot shows the AUC-ROC value of applying the LR algorithm, while the (**b**) plot shows the AUC-ROC value of applying the DT algorithm. The (**c**) plot shows the AUC-ROC value of applying the RF algorithm, and the (**d**) plot shows the AUC-ROC value of applying the NB algorithm. The (**e**) plot shows the AUC-ROC value of applying the KNN algorithm.

When the SMOTE technique is applied, some ML algorithms achieved optimal performance in terms of precision, recall and F-measure. As it is shown in Table 11, the applying of SMOTE technique achieves the best results compared to the previously applied methods (label-encoding and one-hot encoding). The DT, RF and KNN algorithms attain the highest precision, recall and F-measure scores between (98% and 99%). The average AUC-ROC values of applying SMOTE technique are presented in Figure 6.

As presented in Figure 6, the insider threat detection results are improved markedly when applying SMOTE technique. The promising result is obtained by the DT and RF algorithms with an AUC-ROC value of 1, followed by the KNN algorithm with an AUC-ROC value of 0.99. This indicates that we are able to successfully detect the majority of insider data leakage instances. The NB and LR algorithms also obtained good results with AUC-ROC values of 0.84 and 0.79, respectively. In general, out of all utilized evaluation metrics, the SMOTE technique outperforms other applied methods in all utilized ML classifiers. This is due to its effectiveness in improving the performance of the detection by considering and addressing the imbalance data issue before training the ML classifiers.

## 5. Discussion and Comparison

**Figure 7:**
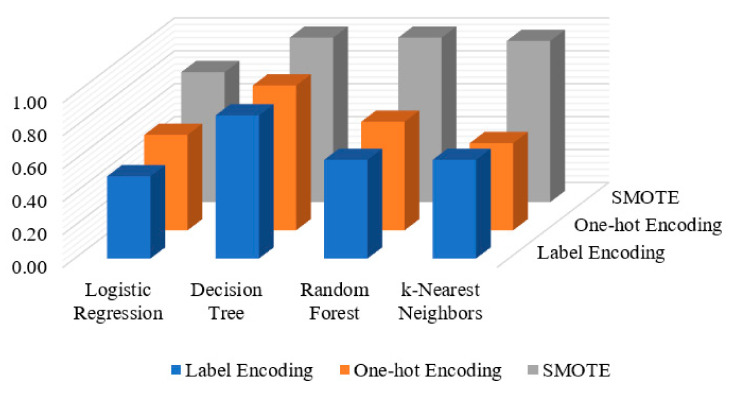
The AUC-ROC curve results of the applied methods (label encoding, one-hot encoding and SMOTE) on different ML algorithms.

The combined results obtained for the three applied methods (label encoding, one-hot encoding and SMOTE) are presented in Figure 7. It is observed that the DT algorithm outperforms other applied ML algorithms, while the LR algorithm obtains the lowest detection results over the three applied methods. With respect to the detection results on various applied methods, SMOTE performs better than other applied methods over all applied ML algorithms, except for LR algorithm which gains a slight increase of 0.08 AUC-ROC value over one-hot encoding method.

We have shown that the insider data leakage detection results of applying the three methods (Label Encoding, One-hot Encoding and SMOTE) in a comparable way. The performance of the applied methods with respect to all the four machine learning classifiers (LR, DT, RF and KNN) is shown in Figure 7. When the label encoding and one-hot encoding methods are utilized, the results show that the detection performance was increased with respect to one-hot encoding method compared to the label encoding method in all the four applied classifiers LR, DT, RF and KNN with AUC-ROC values of 0.58, 0.88, 0.66 and 0.53, respectively. The results depict that the LR, DT, RF and KNN classifiers when applying the SMOTE method are higher than other applied methods with AUC-ROC values of 0.79, 1.0, 1.0, 0.99, respectively. This indicates that the SMOTE is an effective method when it is applied for insider data leakage detection where the utilized dataset is highly imbalanced.

It is noteworthy to compare our approach with related work. In this section, we discuss the most related work to our approach as summarized in Table 12. Since different approaches utilized various metrics and datasets for performance evaluation, we select the papers with an AUC-ROC evaluation metric and those utilized the CERT r4.2 dataset for the aim of comparison. As discussed above, significant factors are overlooked by the related work during the classification modeling (e.g., addressing the imbalanced classes of the dataset and the bias of the encoding process). Therefore, this work differs from previous approaches as it considers the issue of imbalanced dataset and the possible bias issue of the encoding process before proceeding with the classification process. It addresses the imbalanced classes of the dataset to improve the classification at the data level by applying the SMOTE method (Section 4.3). It also considers the bias issue that could occur when some features dominate others during the encoding process. That is by applying the one-hot encoding technique to eliminate the possibility of the bias issue in the matrix of features, for the aim of improving the performance of ML algorithms. It focuses on detecting the insider data leakage threats that could happen during the critical period of an employee lifespan within an organization (before an employee leaves an organization). It applies the most well-known ML algorithms (LR, DT, RF, NB, KNN and KSVM) and assessing them utilizing the appropriate evaluation metrics (precision, recall, F-measure and AUC-ROC value) in order to provide a better insight into the performance of the applied models. Table 12 presents a summary of our approach compared to the related work that are evaluated on a similar dataset (CERT r4.2).

In the comparative analysis of our work with state-of-the-art approaches evaluated on the CERT r4.2 dataset, the well-known ML classifiers produce good and promising results. Table 12 displays the performance of the proposed method compared to existing approaches. Our proposed model demonstrates a clear advantage in the detection performance when compared to other works in the literature for insider threat detection utilizing the CERT r4.2 dataset [73,74,75,76,77,78,79,80,81,82]. The superior detection results are achieved by [80] utilizing HMM and by our work utilizing DT + SMOTE and RF + SMOTE methods with an AUC-ROC value of 1.0, outperforming other previous works. The performance evaluation that is obtained by our method gives more confidence results, as it considers the imbalanced classes of the dataset utilizing the SMOTE technique. When applying the SMOTE using the NB and KNN classifiers, we got the AUC-ROC values of 0.84 and 0.99, respectively. The least detection result is achieved when applying the LR + SMOTE method with an AUC-ROC value of 0.79. It is observed that several works achieved excellent results with AUC-ROC values of 0.98 and 0.99 utilizing different methods such as in [75,79,82]. Our proposed approach achieved an improved AUC-ROC curve value of 1.0 when applying DT+ SMOTE and RF + SMOTE methods. In addition, it considers class imbalance handling of the dataset before training the classifiers, so it can improve the performance of insider data leakage detection effectively.

## 6. Conclusions

Insider data leakage threat is considered an emerging security threat for organizations, especially when it is carried out by malicious insiders before they leave an organization. In this paper, we present a machine learning based model for detecting insider data leakage events in such a threat scenario. The LR, DT, RF, NB, KNN and KSVM machine learning algorithms are trained on the benchmarking CERT dataset to detect insider data leakage events on unseen data. We employ different methods (label-encoding, feature scaling, one-hot encoding and SMOTE) on data granularity level to address possible bias issues caused by an inappropriate encoding process and the imbalanced class problem of the used dataset. The performance of the proposed model is evaluated via a series of experiments in order to determine the best machine learning model utilizing the appropriate evaluation metrics (recall, precision, F-measure and AUC-ROC value). The results show that our model is able to detect insider data leakage events with a high AUC-ROC value, especially when addressing the imbalanced issue of the used dataset by employing SMOTE technique. The experimental results confirm the robustness of the DT and RF machine learning algorithms compared to other applied algorithms, which suggest their benefits under extremely adversarial conditions. Compared with the most related works that are validated on the same dataset, our approach shows a better performance in detecting insider data leakage events during the sensitive period while an insider is planning to commit an attack and leaves the working environment of an organization thereafter. Moreover, the proposed model handles the bias and class imbalance issues that may occur during the encoding and classification processes. We must acknowledge that there is a limitation in the presented work, as well as the related works, which is that they are evaluated on a synthetic dataset, not on a real dataset that can be collected from a real-world organization. However, the validation of insider threat detection models on a real-world environment is still a major challenge in the field of insider threat due to ethical and privacy concerns in the case of revealing real data of an attacked organization to the public. Thus, the future work will include the validation of an insider threat detection system on a real dataset by collecting data of insiders acts throughout systems and networks of a private organization. Doing so will also allow us to evaluate the scalability factor of the insider data leakage system. In the future work, we will also investigate the system under different deep learning algorithms (e.g., recurrent neural networks (RNNs), deep Boltzmann machine (DBM), etc.) to optimize the extraction of higher-level features from the raw data input using multiple layers. In addition, more sophisticated data preprocessing techniques and feature analysis techniques will be utilized to improve the performance of an insider threat detection system on other deep learning algorithms.

## Figures and Tables

**Table 1:** Description of dataset files.

File	Description
Logon	In this file, the logon/off activity of insiders to an organization system is recorded. It contains insiders ids, logon/off events and PCs ids along with the associated timestamps.
File	This file includes the data of insiders’ activities with respect to operations of transferring files to removable devices. The details of file operations are recorded in “file.csv” (e.g., insiders ids, pc ids, file type, content, timestamp, etc.).
HTTP	The http file includes the web browsing logs of insiders. It involves visited URLs, timestamps, insiders’ ids, PCs ids, and some keywords involved in the content of visited webpages.
Email	The email activity information of insiders is stored in the “e-mail.csv” file, such as the ids of insiders, e-mail addresses, timestamps, email size, attachment, etc. Several information can be deduced from the email activity file (e.g., the number of sent emails, whether the recipient is inside or outside an organization, and so on).
Device	The logs of insiders’ activities with respect to the use of removable devices are logged in the “device.csv” file. It includes information of the interactions of insiders with removable devices, such as the devices id, connected, disconnected, timestamp, and so on.

**Table 2:** Data fields and variables of the combined dataset.

Data Field	Variables Sample
Vectors	Logon, logoff, device, http.
Timestamps	mm/dd/yyyy hh:mm:ss AM/PM.
Insider IDs	MAR0955, MCF0600, …
Actions	Connect/Disconnect, Logon/Logoff, http://wikileaks.org
Target	Malicious or non-malicious.

**Table 3:** The extracted dataset features.

Feature	Description
Vector	This feature represents various types of insider activities (logon/logoff of insider sessions, visited webpage, used removable devices).
Timestamp	The timestamps of insiders’ actions are represented in a format of (mm/dd/yyyy hh:mm:ss AM/PM).
Insider ID	The insider ids which are represented as MAR0955, MCF0600, etc.
Action	The actions of insiders that specify the variables of the feature vector (e.g., Connect/Disconnect, Logon/Logoff, visit http://wikileaks.org, etc.).
Target	It represents whether an action of an insider is malicious or not according to the ground truth of the CERT dataset.

**Table 4:** Data type of extracted features.

Feature	Data Type
Vectors	categorical
Timestamps	ordinal
Insider IDs	categorical
Actions	categorical
Target	categorical

**Table 5:** Encoded variables of data features.

Feature	Encoded
Vectors	Int64
Timestamps	Int64
User IDs	Int64
Actions	Int64
Target	Int64

**Table 6:** The employed confusion matrix.

		**DETECTED**	
		**Malicious**	**Non-malicious**
**ACTUAL**	**Malicious**	True positives (TP)	False negatives (FN)
	**Non-malicious**	False positives (FP)	True negatives (TN)

**Table 7:** Size of training set and test set.

Feature Matrix	Training Set	Test Set
2308813	1847050	461763

**Table 8:** Results of applying label encoding and scaling methods on several ML algorithms.

ML Algorithms	Precision		Recall		F-Measure	
	Actual	Scaled	Actual	Scaled	Actual	Scaled
DT	0.67	0.67	0.74	0.74	0.71	0.71
RF	0.60	0.58	0.19	0.19	0.29	0.29
KNN	0.13	0.84	0.01	0.21	0.02	0.32
KSVM	0.00	1.00	0.00	0.15	0.00	0.27

**Table 9:** An example of one-hot encoding process.

Insider Acts	Login	USB	Website	Logoff
Login	1	0	0	0
USB	0	1	0	0
Website	0	0	1	0
Logoff	0	0	0	1

**Table 10:** Results of applying one-hot encoding method on various ML algorithms.

ML Algorithms	Precision	Recall	F-Measure
LR	1.00	0.15	0.26
DT	0.68	0.77	0.72
RF	0.39	0.32	0.35
KNN	0.33	0.06	0.11
KSVM	1.00	0.15	0.27

**Table 11:** Results of applying the SMOTE technique over different ML algorithms.

ML Algorithms	Precision	Recall	F-Measure
LR	0.50	1.00	0.67
DT	0.99	0.99	0.99
RF	0.99	0.99	0.99
NB	0.77	0.95	0.85
KNN	0.98	0.99	0.98

**Table 12:** The performance of the proposed work compared with related work.

Approach	Model	AUC-ROC
Rashid et al. [31]	Hidden Markov models (HMM)	0.83
Al-Mhiqani et al. [73]	Deep neural network (DNN)	0.95
Gamachchi et al. [74]	Attributed graph clustering (AGC)	0.76
Hall et al. [75]	Neural network (NN)	0.95
	Naive Bayesian network (NBN)	0.98
	Support vector machine (SVM)	0.98
	Random forest (RF)	0.88
	Decision tree (DT)	0.93
	Logistic regression (LR)	0.80
Le et al. [76]	Unsupervised ensembles (UE)	0.91
Sharma et al. [77]	Long short-term memory (LSTM)	0.95
Singh et al. [78]	Multi fuzzy classifier (MFC)	0.89
Wang et al. [79]	Principled and Probabilistic Model (PPM)	0.99
Ye et al. [80]	Hidden Markov model (HMM)	1.00
Yuan et al. [81]	Recurrent neural network (RNN)	0.93
	Gated recurrent unit (GRU)	0.91
	Long short-term memory (LSTM)	0.93
Yuan et al. [82]	DT + random	0.90
	Xgboost + random	0.92
	DT + SMOTE	0.98
	Xgboost + SMOTE	0.98
	DT + GAN	0.99
	Xgboost + GAN	0.99
Proposed method	LR + SMOTE	0.79
	DT + SMOTE	1.00
	RF + SMOTE	1.00
	NB + SMOTE	0.84
	KNN + SMOTE	0.99

## Data Availability

The data used to support the findings of this study are available from the corresponding author upon request.
